# Inflammatory memory as a potential driver of rosacea pathogenesis: integrating mechanisms of chronic relapse and perspectives on disease modification

**DOI:** 10.3389/fimmu.2026.1962766

**Published:** 2026-09-30

**Authors:** Ming Zhang, Zhou Zhou, Dandan Zhan, Yumeng Yang, Min Li

**Affiliations:** Department of Dermatology, The General Hospital of Western Theater Command, Chengdu, Sichuan, China

**Keywords:** disease modification, epithelial memory, inflammatory memory, relapse threshold, rosacea, tissue-resident memory T cells, trained immunity

## Abstract

Inflammatory memory refers to functional changes retained by a tissue after an inflammatory episode that allow faster or stronger responses to subsequent challenges. It encompasses epigenetic memory in epithelial and stem cells, trained immunity, and tissue-resident memory T cells. Rosacea is a chronic inflammatory skin disease characterized by recurrent flushing, persistent erythema, and papulopustular lesions, and relapse after treatment cessation is common; the mechanisms that drive recurrence remain unclear. In this hypothesis-generating critical review, we use inflammatory memory as an integrative framework and examine evidence consistent with inflammatory memory in rosacea, including the Toll-like receptor 2/kallikrein-related peptidase 5/cathelicidin antimicrobial peptide (LL-37) positive feedback axis, mast-cell-mediated amplification, neuroimmune amplification, and an interleukin-1β-centered persistent inflammatory program. We draw on mechanistic evidence from psoriasis and atopic dermatitis related to tissue-resident memory T cells, epithelial memory, and trained immunity, while explicitly separating established rosacea-specific observations from indirect cross-disease parallels and speculative claims. A multilayer memory network model is proposed in which repeated triggers write memory and lower the relapse threshold; disease modification is then discussed as a candidate therapeutic goal, together with potential strategies and testable hypotheses. Direct molecular evidence for inflammatory memory in rosacea is still lacking, alternative mechanisms remain plausible, and disease modification should be viewed as a perspective rather than an established endpoint.

## 1 Introduction

Rosacea is a chronic inflammatory skin disease characterized by centrofacial flushing, persistent erythema, telangiectasia, and papulopustular lesions. Patients experience repeated episodes, and relapse after treatment cessation often occurs within weeks to months. In a phase III randomized controlled trial (RCT), patients with papulopustular rosacea who had responded to 16 weeks of treatment were followed after withdrawal; the median time to relapse was 115 days with ivermectin 1% cream and 85 days with metronidazole 0.75% cream (1). In a retrospective comparative study, the 24-week no-relapse rate was 48.6% when oral metronidazole was added to oral minocycline, compared with 18.1% with minocycline alone (2). Relapse also remains difficult to prevent even during maintenance therapy: the relapse rate during 40 weeks of maintenance with subantibiotic-dose doxycycline (40 mg once daily) was 13.8%, significantly lower than the 27.7% in the control group (3); after treatment with 540 nm intense pulsed light, the recurrence rate at 2 years of follow-up was 8.41%, compared with 48.33% without long-term intervention (4).

Current guidelines recommend phenotype-based symptomatic treatment (5), but available therapies mainly control active inflammation, and no disease-modifying approach exists. Clinically, the same triggers, such as temperature changes, ultraviolet exposure, spicy food, and emotional stress, can repeatedly induce episodes, and some patients become increasingly sensitive to stimulation over time. This pattern suggests that rosacea-affected skin does not return completely to a resting state but instead retains an inflammatory condition shaped by prior episodes. Concepts such as trained immunity, epithelial inflammatory memory, and tissue-resident memory T cells (TRM) offer a framework for understanding recurrence in chronic inflammatory skin diseases. In this review, we use this framework to integrate the evidence on chronic relapse in rosacea and to discuss disease modification as a candidate therapeutic goal. Throughout the review, we distinguish three evidence levels: established rosacea-specific observations, indirect evidence from other diseases or models, and speculative hypotheses that require direct testing. We also explicitly discuss alternative explanations and contradictory findings and state what evidence would falsify the proposed model.

## 2 Inflammatory memory: conceptual framework and evidence boundaries

Inflammatory memory is a functional concept: after an inflammatory episode, a tissue does not return fully to baseline and instead responds faster or more strongly to subsequent stimulation. Based on the cell types that carry the memory, we classify inflammatory memory into three categories: trained immunity, epithelial/non-immune cell inflammatory memory, and TRM.

Trained immunity refers to the nonspecific enhancement of responsiveness that innate immune cells, such as monocytes and macrophages, acquire after an initial stimulus. The concept was first systematically described in infection immunology, and its core mechanisms are epigenetic and metabolic reprogramming (6, 7). Epigenetic changes include histone H3 lysine 4 trimethylation (H3K4me3) and histone H3 lysine 27 acetylation (H3K27ac). Training does not always have harmful consequences: macrophages trained with low-dose lipopolysaccharide (LPS) attenuated systemic sclerosis-like fibrosis, whereas macrophages trained with Bacillus Calmette-Guérin (BCG) worsened fibrosis, and adoptive transfer reproduced the corresponding effects (8). Thus, the direction and intensity of training determine its pathological outcome.

Epithelial/non-immune cell inflammatory memory refers to the ability of structural cells to retain the effects of prior inflammation. In airway diseases such as chronic rhinosinusitis with nasal polyps, human nasal epithelial progenitor cells exposed to allergic inflammation, specifically interleukin-4 (IL-4)/interleukin-13 (IL-13), retained transcriptional memory. In one patient with concomitant severe atopic dermatitis (AD), treatment with an anti-interleukin-4 receptor α (IL-4Rα) antibody partially reset the basal cell state of nasal polyps (9).

In mouse epidermal stem cells, inflammatory memory is acquired in a stimulus-specific manner and is established by the coordinated action of the broadly acting stress-responsive transcription factors FOS and JUN together with signal transducer and activator of transcription 3 (STAT3). During establishment, these factors open the chromatin of memory domains; during maintenance, stem-cell transcription factors and histone-modifying enzymes keep the domains accessible. On secondary stimulation, such as full-thickness wounding, FOS rapidly re-enters memory domains and reactivates transcription, suggesting a role for this memory in tissue repair (10).

Fibroblast-like synoviocytes from patients with rheumatoid arthritis retain gene-specific chromatin-level memory after tumor necrosis factor (TNF) withdrawal and can amplify interferon responses (11). These findings show that long-term memory is not restricted to immune cells; structural cells can also retain it.

TRM are the skin-resident form of adaptive immune memory and can persist locally for long periods (12). Clinically resolved psoriatic lesions retain CD49a^+^/CD103^+^ epidermal CD8^+^ TRM and type 22 helper T (Th22)/type 17 cytotoxic T (Tc17) cells that can still secrete interleukin-17 (IL-17)/interleukin-22 (IL-22) 6 years after treatment cessation (13). Resolved lesions also contain oligoclonally expanded pathogenic IL-17^+^ αβ T cells (14). The IL-17A-dominated tissue response elicited by activation of these resident T cells can stratify the risk of relapse after withdrawal of ultraviolet phototherapy (15). In chronic skin diseases, these three categories of memory have increasingly been integrated into a unified framework of inflammatory memory (16).

The evidence above comes mainly from infection immunology, tissue repair, psoriasis, AD, airway disease, and rheumatoid arthritis; no direct molecular evidence for the three inflammatory memory layers in rosacea exists. These mechanisms can therefore serve only as analogies for generating testable hypotheses and cannot be taken as evidence that the same mechanisms operate in rosacea.

### 2.1 Evidence hierarchy used in this review

To avoid overstating the strength of the current literature, we classify each claim as established, indirect, or speculative. Established claims are supported by rosacea-specific primary data, including randomized clinical trials (RCTs), cohorts, human tissue analyses, or functional experiments in relevant rosacea models. Indirect claims are supported by mechanistic studies in other inflammatory skin diseases or by ex vivo and animal models that have not been confirmed longitudinally in patients. Speculative claims are reasonable extensions of the framework for which no direct rosacea data exist. Where possible, we state explicitly what additional observation would support or refute a given claim. This hierarchy is used throughout the review and is summarized for the main model components in Section 9.

## 3 Evidence consistent with inflammatory memory in rosacea

Although direct molecular evidence of inflammatory memory in rosacea is lacking, several clinical and mechanistic features are consistent with memory-like behavior: a self-sustaining inflammatory axis, a stereotyped time course of relapse after treatment withdrawal, and a re-primable state in non-lesional skin.

### 3.1 The KLK5/LL-37 positive feedback axis: a self-sustaining inflammatory program

The candidate mechanism closest to inflammatory memory in rosacea is a self-sustaining positive feedback axis. The axis begins with Toll-like receptor 2 (TLR2): TLR2 expression is markedly increased in the epidermis of rosacea but not in AD or psoriasis, and keratinocyte TLR2 overexpression promotes kallikrein-related peptidase 5 (KLK5) release through a calcium-dependent pathway (17). This may explain why patients respond strongly to environmental stimuli. KLK5 is a serine protease that processes the cathelicidin precursor into the cathelicidin antimicrobial peptide (LL-37), which in turn activates inflammatory responses; these steps were first established in skin and keratinocytes from patients with rosacea (18).

Temperature changes, ultraviolet exposure, microbial products, and dietary factors can repeatedly activate the same pathway, which offers a molecular explanation for why the same triggers cause repeated episodes at the same site.

The axis is not only an initiating step of inflammation but also a common target of existing treatments. Doxycycline does not directly inhibit serine proteases; instead, it reduces KLK5 activation and LL-37 generation indirectly by inhibiting matrix metalloproteinases (MMP) (19), which may explain the long-term efficacy of subantibiotic-dose doxycycline. Treatment with azelaic acid 15% gel for 16 weeks reduced cathelicidin and KLK5 expression and serine protease activity (SPA) in lesional skin, with TLR2 suppression observed in mouse skin (20). A small RCT showed that a topical serine protease inhibitor lowered SPA and that the reduction correlated with improvement in severity (21).

Mast cells are key effectors of this axis. After intradermal injection of LL-37, mast-cell-deficient mice do not develop rosacea-like changes; mast-cell stabilizers downregulate matrix metalloproteinase 9 (MMP9) and C-X-C motif chemokine ligand 2 (CXCL2); and in patients with erythematotelangiectatic rosacea, 8 weeks of topical cromoglycate reduced MMP activity in lesions (22). Recent mechanistic studies have identified several receptors through which LL-37 activates mast cells: TLR2 with downstream Janus kinase 2 (JAK2)/STAT3 signaling (23), the Mas-related G protein-coupled receptor X2 (MRGPRX2) in humans and its mouse ortholog, Mas-related G protein-coupled receptor B2 (MRGPRB2) (24, 25), and mitochondrial DNA release with cyclic GMP-AMP synthase/stimulator of interferon genes (cGAS/STING) pathway activation (26). Mast cells therefore serve as both an amplification hub and a potential intervention node. Mast-cell-derived mediators also act on vascular endothelium and sensory nerves, linking immune amplification to neurovascular symptoms such as flushing and burning (22, 27, 28). In this way, the axis and neurogenic inflammation reinforce each other and jointly contribute to the chronicity network.

At the same time, the causal position of mast cells should not be overstated. In a comparative histopathological study of 20 Korean patients, lesional inflammatory mediator expression did not differ significantly between mast cell-rich and mast cell-poor rosacea skin (interleukin-6 (IL-6), P = 0.519; LL-37, P = 0.443; TNF-α, P = 0.400) (29). Mast cells may thus be an important amplifier rather than a sufficient or universal determinant of lesional inflammation, and their contribution may vary across subtypes and disease phases.

Neuroimmune interactions may be an important component of the sensitized state. Sensory neurons contribute to flushing and neurogenic inflammation through transient receptor potential vanilloid 1 (TRPV1) and other ion channels; TRPV1-nerve growth factor (NGF)-tropomyosin receptor kinase A (TrkA) signaling is activated in rosacea skin and is further exacerbated by Demodex mites (27). In a mouse model, high-sensitivity sensory neurons released calcitonin gene-related peptide (CGRP), which directly activated receptor activity-modifying protein 1 (RAMP1)-expressing γδ T cells to produce IL-17A; γδ T cells were increased in rosacea lesions, and genetic depletion of γδ T cells or CGRP receptor antagonism reduced rosacea-like dermatitis (30). In patients, serum CGRP levels differed between neurogenic and non-neurogenic rosacea, supporting a clinically relevant neurogenic endotype (28). Peripheral blood from 280 patients with rosacea showed elevated interleukin-1β (IL-1β), IL-17, and interleukin-8 (IL-8) that correlated positively with anxiety and depression (31). These observations link neural, innate, and adaptive components into a recurrent activation circuit, but whether the neuroimmune loop itself stores memory or merely amplifies pre-existing memory remains unknown. Similarly, whether mast cells themselves acquire a long-lived primed state after repeated LL-37 stimulation, analogous to trained immunity in macrophages, remains untested and warrants direct functional study.

More direct evidence for persistence comes from tissue-level omics studies. A paired transcriptomic and proteomic analysis of lesional and non-lesional tissue from five patients with papulopustular rosacea showed significant upregulation of mitogen-activated protein kinase (MAPK) and TNF pathways centered on IL-1β. Non-lesional tissue explants challenged with IL-1β recapitulated the lesional profile (32), suggesting that the inflammatory program of lesional skin may pre-exist in normal-appearing skin and can be re-primed by a single cytokine. This pattern is consistent with a “program + trigger” memory-like model. However, the sample size was small, the experiment was ex vivo, and the exogenous IL-1β concentration may not reflect physiological levels. These results suggest, but do not prove, that a re-primable inflammatory program exists in non-lesional skin. Integrated proteomic, phosphoproteomic, and transcriptomic analyses further support the coexistence of immune-inflammatory and neurovascular programs in rosacea skin (33, 34).

In summary, the TLR2→KLK5→LL-37→mast cell/IL-1β axis has positive-feedback and self-maintaining features and is the most plausible candidate for an inflammatory memory-like state in rosacea. These features may explain why the same triggers repeatedly induce episodes at the same site; however, whether the pathway persists in a residual state between episodes remains unsupported by longitudinal molecular evidence. The evidence currently remains at the level of molecular pathways, and direct verification through human lesional epigenomic or single-cell studies is still lacking.

### 3.2 Relapse after treatment withdrawal: treatment-dependent remission and relapse kinetics

Relapse after treatment withdrawal is a relatively consistent memory-like phenomenon in clinical studies of rosacea. Multiple RCTs and cohort studies show that maintenance therapy reduces relapse rates, whereas relapse after withdrawal occurs within weeks to months (1–4). This indicates that remission depends largely on continuous suppression of inflammation rather than on true resetting of tissue programs. The time to relapse after withdrawal is quantifiable and reproducible across studies, making it a candidate observational metric for disease-modification endpoints. This clinical pattern is consistent with a locally retained, reactivatable inflammatory program, but it does not by itself prove the existence of tissue memory; current therapies still aim mainly at controlling active symptoms.

Most existing relapse assessments rely on physician-assessed lesion recurrence and pay insufficient attention to subjective symptoms such as flushing and burning or to changes in trigger thresholds; predictive factors for relapse have not been established. In a prospective study of 60 patients with papulopustular rosacea treated with ivermectin 1% cream for 16 weeks, 70% (42/60) achieved an Investigator’s Global Assessment (IGA) score of 0. During post-withdrawal follow-up of up to 32 weeks, the median time to first relapse was 140 days (range 84–224 days; mean 152.44 days), and the relapse rate during follow-up was 45.24%, with most relapses being mild (IGA 1) (35). This study used relapse kinetics as its main outcome and shows that the time to relapse after withdrawal is quantifiable and can serve as a candidate disease-course endpoint; however, it was a single-arm observational study with a small sample and did not establish predictive factors for relapse. A real-world Japanese cohort of 42 patients followed for 1 year further showed that physician- and patient-reported severity measures declined over time, indicating that longitudinal disease-course outcomes are feasible in routine practice (36).

### 3.3 Non-lesional “program-trigger” evidence and relapse threshold indicators

Non-lesional tissue studies provide preliminary evidence that the memory extends beyond lesional boundaries. Non-lesional tissue explants can be re-primed by IL-1β into a lesional-like state (32). TLR2 overexpression in lesional epidermis (17) further points to enhanced keratinocyte responsiveness to environmental stimuli, although it does not establish that non-lesional skin shares this phenotype.

Trigger-burden assessment is a practical way to quantify this threshold clinically. In a prospective case-control study of 100 patients with rosacea and 100 controls, hot bathing habits were significantly more common in the rosacea group (P = 0.011), and heat, sunlight, emotional stress, alcohol, and spicy food were the most frequently reported triggers (37). Standardized trigger diaries or trigger-burden scores could therefore be used to track whether the minimum stimulus intensity required to induce an episode decreases over time, a prediction that follows directly from the memory model (Section 5).

## 4 Cross-disease mechanistic evidence for drivers of recurrence

Because direct evidence in rosacea is limited, mechanistic studies from other chronic skin diseases help clarify how the three categories of inflammatory memory may drive recurrence. These studies provide analogies and design templates, not proof of the same mechanisms in rosacea.

### 4.1 The TRM layer

Psoriasis provides relatively complete TRM evidence for same-site recurrence. Epidermal CD8^+^ TRM and Th22/Tc17 cells persist long-term in resolved lesions and can secrete pathogenic factors (13); the pathogenic T cells are present as oligoclonally expanded clones (14); and the IL-17A-dominated response elicited by resident T-cell activation predicts early relapse after withdrawal of phototherapy (15). These results indicate that even after complete clinical resolution, pathogenic T cells can remain resident and drive recurrence at the same site.

Pathogenic clones are also found in non-lesional skin (14), indicating that memory T cells can pre-exist in clinically normal areas. Once reactivated by environmental or local factors, they may contribute to the formation of new or recurrent lesions.

In rosacea, direct evidence for skin-resident T cells has only recently emerged. A single-cell RNA sequencing study of 131, 243 cells from nine patients with rosacea and three healthy controls identified increases in type 1/type 17 T cells and skin-resident memory T cells, the latter expressing ITGAE, CD69, and CD103 (38). An immunohistochemical study also showed altered CD4/CD8 T-cell ratios and CD123^+^ plasmacytoid dendritic cells in rosacea compared with discoid lupus erythematosus and polymorphous light eruption (39). Two important caveats apply. First, T-cell infiltrates in rosacea are generally sparse and less dense than in psoriasis, so the demonstration of resident memory populations does not establish that they drive relapse. Second, these data are cross-sectional; the cells could be bystanders recruited by ongoing inflammation rather than the memory carriers of the model. Whether resident-cell abundance is associated with relapse risk in rosacea remains unknown. Functional studies, such as quantification of clonal expansion, reactivation-induced IL-17A release from sorted resident cells, and longitudinal correlation of resident-cell abundance with time to relapse, are needed before the TRM layer can be accepted in rosacea.

### 4.2 The epithelial/non-immune cell memory layer

Epithelial cells can also retain disease memory. Epidermal keratinocytes isolated from resolved psoriatic lesions retain disease-related transcriptomic features and 5-methylcytosine (5-mC)/5-hydroxymethylcytosine (5-hmC) epigenetic changes, with enrichment of WNT/TNF/mammalian target of rapamycin (mTOR) pathways (40). Mechanistic studies of airway epithelial progenitors (9), epidermal stem cells (10), and synovial fibroblasts (11) further indicate that inflammation can write reactivatable chromatin programs into non-immune cells.

Epithelial memory is not an irreversible all-or-nothing switch. Airway epithelial progenitors maintain transcriptional memory after IL-4/IL-13 withdrawal (9), showing that such memory can be retained long-term, and epidermal stem cells remain in a poised state through long-term open chromatin regions (10). Interferon-γ (IFN-γ)-induced transcriptional memory further shows that establishment and maintenance can be controlled by different mechanisms: establishment requires signal transducer and activator of transcription 1 (STAT1), whereas maintenance is STAT1-independent, and the memory is inherited through cell division as chromatin changes and can be reversed (41). Establishment, maintenance, and recall can therefore be targeted separately, which provides a molecular basis for staged therapy.

### 4.3 The trained immunity layer

Trained immunity can either promote or inhibit tissue damage depending on the direction of the response (8). Repeated TLR2/LL-37 stimulation in rosacea could theoretically retrain local macrophages, and a mechanistic study in mice showed that guanylate-binding protein 5 (GBP5) promotes rosacea-like inflammation by skewing macrophage polarization toward the M1 phenotype through the NF-κB pathway (42). However, direct evidence in human skin innate immune cells remains lacking.

The same macrophage population can differentiate toward pro-fibrotic or anti-fibrotic phenotypes under different training conditions, and the phenotype can be retained after adoptive transfer (8). If trained immunity operates in rosacea, the therapeutic goal would be to steer it rather than simply deplete it, although this remains a conceptual inference at present.

### 4.4 Cellular and molecular basis of relapse after treatment withdrawal

Drug clearance is an expected component of relapse after treatment withdrawal, but the reproducible timing of relapse and the re-emergence of specific inflammatory programs suggest that withdrawal relapse is not simply passive restoration of baseline inflammation. After withdrawal of an anti-CD11a monoclonal antibody, relapsing psoriatic lesions show T-cell reinfiltration and increases in inflammatory myeloid dendritic cells expressing TNF/inducible nitric oxide synthase (iNOS)/TNF-related apoptosis-inducing ligand (TRAIL) (43). After withdrawal of the interleukin-23 (IL-23) inhibitor guselkumab, serum IL-17A/F and IL-22 rise at the time of relapse, with the cytokine increase lagging behind the clinical recurrence (44). Molecular programs are thus reactivated at the time of relapse; whether tissue molecular changes precede clinically detectable recurrence remains to be tested in longitudinal studies.

A 12-year multicenter cohort of 991 treatment episodes further showed that each additional prior treatment withdrawal increased the risk of relapse by approximately 23%, and that the speed of relapse differed across biologics with different mechanisms (45). These findings suggest that repeated withdrawal-relapse cycles may accumulate a relapse-prone state.

Withdrawal-based molecular monitoring cohorts could therefore be conducted in rosacea, with serial measurements of the LL-37/IL-1β axis and neurovascular markers before clinical relapse, to test the hypothesis that tissue programs are rebuilt before relapse becomes clinically apparent.

## 5 A hypothetical multilayer memory network model

Based on the evidence above, we propose a hypothetical integrated model (**Figure 1**). External triggers first activate the TLR2/KLK5/LL-37 axis, and inflammation is then amplified through mast cells and IL-1β (17, 19, 22, 32). Repeated episodes write multilayer memory into the skin: epigenetic imprints in keratinocytes and stem cells (10, 40), directional training of macrophages (8, 42), local residency of pathogenic T cells (13–15, 38), and fibroblast remodeling (11). Nerve-ending sensitization and changes in ion-channel activity are included as a memory-adjacent amplifier rather than as direct evidence of inflammatory memory (27, 28, 30, 31). These memories jointly lower the relapse threshold, which clinically manifests as treatment-dependent remission (1–4).

**Figure 1:**
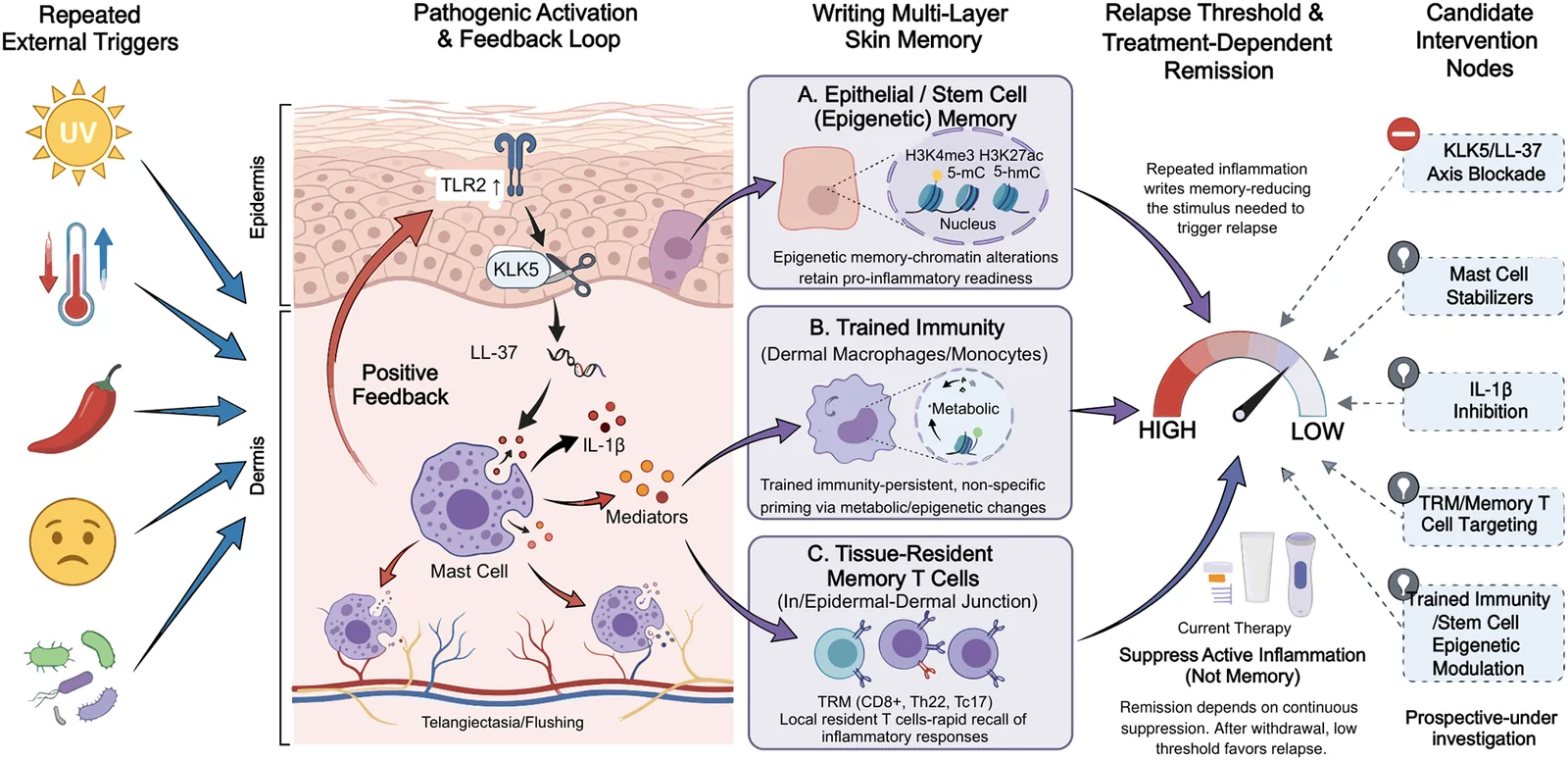
Hypothetical multi-layer inflammatory memory model underlying chronic relapse in rosacea. Repeated triggers activate the TLR2→KLK5→LL-37 positive feedback axis, which is amplified through mast cells and IL-1β. Recurrent inflammation progressively writes persistent memory at multiple skin layers, including candidate epigenetic marks (H3K4me3/H3K27ac; 5-mC/5-hmC), trained immunity in dermal macrophages/monocytes, and tissue-resident memory T cells. These memory layers lower the relapse threshold, making remission dependent on ongoing treatment. Current therapies suppress active inflammation but do not erase memory; candidate interventions targeting each node are prospective and require validation. Abbreviations: 5-mC, 5-methylcytosine; 5-hmC, 5-hydroxymethylcytosine; H3K4me3, histone H3 lysine 4 trimethylation; H3K27ac, histone H3 lysine 27 acetylation; IL-1β, interleukin-1β; KLK5, kallikrein-related peptidase 5; LL-37, active fragment of cathelicidin antimicrobial peptide; Tc17, type 17 cytotoxic T cell; Th22, type 22 helper T cell; TLR2, Toll-like receptor 2; TRM, tissue-resident memory T cell.

The core prediction of the model is a lowered relapse threshold: the stimulus intensity required to induce an episode decreases with repeated inflammation. Repeated inflammation leaves the skin with low-level background activation during clinical quiescence, so that previously tolerated stimuli can cross the episode threshold. Treatment suppresses active inflammation and maintains clinical remission but may not erase memory traces; after withdrawal, the threshold therefore quickly returns to a level at which episodes occur.

The model rests on three pillars supported by different types of evidence. First, the “program + trigger” concept is supported by direct mechanistic evidence in rosacea: non-lesional tissue explants can be re-primed by IL-1β into a lesional-like state (32). Second, withdrawal studies show that molecular programs are reactivated at the time of relapse: relapsing psoriatic lesions show T-cell reinfiltration and inflammatory dendritic-cell activation (43), and serum cytokines rise at the time of relapse rather than before it (44); whether molecular changes precede clinically detectable recurrence remains untested. Third, the idea that repeated relapse accumulates susceptibility derives from a psoriasis cohort, in which more prior treatment withdrawals were associated with higher relapse risk (45). Direct evidence in rosacea currently covers only the first pillar and is limited to a small ex vivo study; the complete multilayer memory network should therefore remain a hypothesis to be tested.

To make the model testable, we propose an operational definition of the relapse threshold. We define the relapse threshold as the minimum stimulus intensity, or the cumulative trigger burden, required to induce a clinical episode in a given patient. Trigger burden can be measured with a standardized diary that records exposure to heat, ultraviolet light, alcohol, spicy food, emotional stress, and other reported triggers, with each exposure weighted by intensity and duration (37); objective provocation protocols could complement diary data in research settings. A lowered threshold would then manifest as one or more of the following: a shorter time to relapse after withdrawal, relapse after milder or fewer triggers, and a greater cumulative number or severity of episodes per unit time. A meaningful threshold shift should ultimately be calibrated against the minimal clinically important difference of validated rosacea severity measures and against prespecified disease-course changes (for example, a prolongation of relapse-free time or a reduction in trigger burden exceeding the measurement error of the diary), with replication in an independent cohort; we deliberately do not assign a numeric value at this stage because no calibration data exist in rosacea.

A further testable feature of the model is its hierarchical structure. If epithelial/stem-cell memory forms the long-term layer, trained immunity the intermediate layer, and TRM the local rapid effector layer, different therapeutic strategies should act on different layers. This ordering does not imply equal evidence weight: in rosacea, direct support is currently strongest for the re-primable non-lesional program (32), whereas the TRM layer is the least supported and requires functional and longitudinal validation (38). Combination or sequential therapy may come closer than single-target therapy to the goal of rewriting memory.

## 6 Disease modification: from symptom control to altering disease course

Disease modification refers to early intervention that alters the natural history of a disease so that benefit persists after treatment withdrawal (46). In inflammatory skin diseases, the key evidence for disease modification is sustained remission after withdrawal at a time when the drug has largely been cleared from the body (46, 47).

The distinction between true disease modification and prolonged pharmacological effect is therefore central. We propose that a disease-modification claim in rosacea requires (1): a randomized withdrawal design in which patients are allocated to continue treatment or switch to placebo after a defined induction phase (2); pharmacokinetic confirmation that the drug has fallen below the limit of quantification, or that the washout period exceeds at least five drug half-lives, before off-therapy outcomes are assessed; and (3) prespecified disease-course endpoints, including relapse-free survival, time to first relapse, cumulative relapse burden, and changes in trigger threshold. For topical agents whose systemic absorption is negligible, the pharmacokinetic criterion should be adapted to local drug delivery data or a sufficiently long off-treatment observation period rather than systemic half-life thresholds. Without these elements, durable benefit after stopping treatment cannot be separated from residual drug effect. No rosacea trial has yet met these criteria, and currently available rosacea RCTs should therefore be read as evidence of efficacy during or immediately after treatment rather than of disease modification.

Most existing rosacea RCTs support the first half of this distinction: they show efficacy during active treatment but do not include post-withdrawal follow-up. In the two phase 3 trials of oral DFD-29 (low-dose minocycline), 16 weeks of treatment produced higher IGA success rates than placebo (32.9% and 34.1% vs 12.0% and 14.2%, respectively) and greater reductions in inflammatory lesions (between-group differences of 9.2 and 6.8 lesions), but no off-therapy phase was reported (48). Similarly, the phenotype-based Cochrane review of 152 studies involving 20, 944 participants found high- or moderate-quality evidence for brimonidine, azelaic acid, ivermectin, and doxycycline 40 mg, with only 16 of 152 studies judged at low risk of bias (49). The German S2k guideline likewise summarizes current phenotype-based treatment but does not define disease-modification endpoints (50). A recent network meta-analysis across phenotype-relevant outcomes provides a broader comparative basis for trial planning (51), yet the field still lacks randomized withdrawal studies that could support a disease-modification claim.

Precedents from AD show how such trials can be designed. In AD, off-therapy or reduced-dosing studies have demonstrated sustained control after stopping or spacing treatment (52, 53), and trials of anti-OX40 (CD134) and anti-OX40 ligand (OX40L) antibodies, which target T-cell memory pathways, showed durable benefit after a limited treatment course (54, 55). Early intervention in infant AD reduced subsequent sensitization and atopic march outcomes (56–58). Together, these lines of evidence show that disease modification does not require all patients to remain relapse-free after withdrawal; rather, early, adequate, or memory-targeted therapy can increase the probability of sustained off-therapy remission and reduce the severity and speed of relapse.

In rosacea, existing RCTs mostly use short-term efficacy or maintenance-phase relapse as endpoints (1–4), and no clinical trial has yet targeted disease modification. Candidate endpoints could include relapse-free duration after withdrawal, cumulative relapse burden, trigger threshold (the dose or number of stimuli required to induce an episode), duration of resolution of persistent erythema, and markers of inflammatory load and tissue remodeling such as LL-37, KLK5, MMP, and IL-1β. Given that time to relapse after withdrawal is already quantifiable in rosacea studies (1, 3, 35, 48), upgrading relapse time to a formal endpoint and adding trigger-burden and subjective-symptom records is a feasible path toward disease-modification research.

In a retrospective cohort with relapse after treatment withdrawal as the endpoint, 107 patients with rosacea who had responded to oral minocycline (100 mg/day) were analyzed; 49 also received 2–4 sessions of 595 nm pulsed dye laser (PDL). During 1 year of post-withdrawal follow-up, the overall relapse rate was 42.1% (45/107); it was 48.3% (28/58) in the minocycline monotherapy group and 34.7% (17/49) in the combination group. Cox analysis identified PDL as a protective factor for relapse (hazard ratio (HR) = 0.492, 95% confidence interval (CI) 0.257-0.941, P = 0.032) (59). These findings suggest that combination therapy may prolong the relapse-free period after withdrawal, but the retrospective design and baseline differences between groups require prospective validation. This cohort should therefore be read as evidence that long follow-up and a combined pharmacologic-laser strategy are feasible, not as demonstration of disease modification.

## 7 Candidate strategies and evidence levels

Based on the available evidence, candidate strategies can be grouped into six levels, as shown in **Table 1** (1): interrupting the KLK5/LL-37 positive feedback axis, supported by doxycycline (19), azelaic acid (20), and topical serine protease inhibitors (21), with the highest translational feasibility (2); stabilizing mast cells, supported by topical cromoglycate (22), MRGPRX2/MRGPRB2 antagonism (24), and TLR2/JAK2/STAT3 signaling blockade (23) in experimental models (3); blocking the IL-1β axis, supported by NOD-like receptor family pyrin domain-containing protein 3 (NLRP3)-dependent LL-37 models (60, 61) and human skin mechanistic evidence (32), and requiring interventional studies (4); targeting T-cell/memory pathways, supported by the off-therapy benefits of anti-OX40/OX40L in AD (54, 55) by analogy, with IL-17 and JAK-STAT inhibition showing preliminary clinical signals in rosacea (62, 63) (5); early intensive or sequential therapy, supported by early-intervention evidence in AD (56–58), with no direct data in rosacea; and (6) epigenetic reprogramming and steering of trained immunity, informed by the directionality of trained immunity (8), the establishment, maintenance, and recall of epidermal stem-cell memory (10), and the reversibility of transcriptional memory (41), currently a conceptual outlook.

**Table 1:** Candidate disease-modifying strategies in rosacea and current evidence levels.

Strategy	Representative evidence	Evidence type	Main gap
Interrupting the KLK5/LL-37 positive feedback axis	Doxycycline, azelaic acid, SPA inhibitors (19–21)	Mechanism plus small clinical studies	No RCTs with withdrawal-relapse or epigenetic-resetting endpoints
Stabilizing mast cells	Topical cromoglycate (22); MRGPRX2/MRGPRB2 antagonists (24); TLR2/JAK2/STAT3 blockade (23)	Human skin mechanism plus experimental models	No rosacea efficacy-endpoint RCT; negative mast-cell-density study (29)
Blocking the IL-1β axis	NLRP3-dependent LL-37 models (60, 61); lesional transcriptome-proteome (32)	Preclinical mechanism plus human skin mechanism	No clinical intervention data
Targeting T-cell/memory pathways	Anti-OX40/OX40L (54, 55); secukinumab and tofacitinib (62, 63)	RCTs in other diseases plus small rosacea studies	No memory-focused rosacea RCT; TRM role unproven (38)
Early intensive/sequential therapy	Early-intervention trials in AD (56–58)	RCTs/cohorts in other diseases	No early-intervention data in rosacea
Epigenetic reprogramming/steering trained immunity	Establishment-maintenance-recall studies (10, 41); trained immunity directionality (8)	Preclinical mechanism	No rosacea epigenomic evidence; conceptual outlook only

AD, atopic dermatitis; IL-1β, interleukin-1β; JAK, Janus kinase; KLK5, kallikrein-related peptidase 5; LL-37, active fragment of cathelicidin antimicrobial peptide; MRGPRX2, Mas-related G protein-coupled receptor X2; MRGPRB2, mouse Mas-related G protein-coupled receptor B2; NLRP3, NOD-like receptor family pyrin domain-containing protein 3; OX40, CD134; OX40L, OX40 ligand; RCT, randomized controlled trial; SPA, serine protease activity; STAT3, signal transducer and activator of transcription 3; TLR2, Toll-like receptor 2; TRM, tissue-resident memory T cell.

This hierarchy reflects the completeness of the evidence chain rather than a formal grading system. The more directly a strategy is supported by rosacea-specific mechanistic and clinical data, the higher its translational feasibility; strategies validated only in other diseases can guide research but do not constitute established therapeutic evidence.

NLRP3/IL-1β is an important downstream effector node. LL-37 drives rosacea-like skin inflammation in an NLRP3-dependent manner in mice (60), and supramolecular salicylic acid ameliorates LL-37-induced rosacea-like eruptions by suppressing NLRP3-mediated inflammasome activation (61). Experience with NLRP3 inhibitors in autoinflammatory diseases may inform targeted strategies in rosacea, but no rosacea-specific interventional data exist.

The relative disease-modification value of early intensive therapy versus long-term maintenance therapy has not been distinguished in rosacea. Trials comparing early adequate control with on-demand symptomatic treatment for their effects on time to relapse after withdrawal are needed.

Original clinical evidence for targeted therapy in rosacea remains limited. An exploratory, open-label, investigator-initiated trial of the IL-17A inhibitor secukinumab in moderate-to-severe papulopustular rosacea showed significant improvement in inflammatory lesions, overall severity, and quality of life (62). In a retrospective case series, 21 patients with erythematotelangiectatic or papulopustular rosacea treated with the Janus kinase (JAK) inhibitor tofacitinib 5 mg (alone or combined) achieved IGA ≤ 1 in 15 cases (71.4%), with a mean IGA decrease of 2.24 (63). These results suggest that IL-17 and JAK-STAT pathways may be candidate nodes for targeted therapy and disease-modification research in rosacea, but the evidence is limited to small, open-label, or retrospective studies and is insufficient to support a disease-modification conclusion.

## 8 Challenges, testable hypotheses, and future directions

Translating the inflammatory memory framework to rosacea faces five challenges. First, direct molecular evidence of inflammatory memory in lesional and non-lesional tissue is lacking. No published study has directly demonstrated H3K4me3, H3K27ac, or 5-mC/5-hmC changes in rosacea skin, so we explicitly identify this as the central evidence gap rather than implying that such marks have been shown. Baseline studies using chromatin accessibility analysis, single-cell epigenomics, histone modification profiling, and methylation sequencing are needed. Second, relapse threshold and cumulative relapse burden lack standardized definitions; although time to relapse after withdrawal is quantifiable (1, 3, 35), no unified method exists for measuring trigger burden. Third, subtype and phenotype heterogeneity may correspond to different memory networks: erythematotelangiectatic rosacea is dominated by neurovascular sensitization (27, 28, 30, 31), whereas papulopustular rosacea is dominated by immune-inflammatory programs (32, 34), so stratified studies are required. Fourth, disease-modification endpoints require longer follow-up, which conflicts with conventional RCT timelines. Fifth, most relapse studies observe a short maintenance period or a single withdrawal event and lack disease-course dimensions such as cumulative relapse count, relapse severity, and quality of life, which may underestimate relapse burden.

To distinguish memory marks from general inflammatory changes in future studies, we propose four criteria (1): persistence in non-lesional or clinically resolved skin during remission, when active inflammation is absent (2); enrichment at functional regulatory regions, such as promoters and enhancers, rather than a genome-wide inflammatory footprint (3); association with subsequent relapse or with the response to standardized re-stimulation; and (4) functional validation, meaning that genetic deletion or pharmacological reversal of the mark changes the relapse phenotype. These criteria should guide the design of longitudinal chromatin and methylation studies and prevent over-interpretation of transient inflammatory signals as memory.

Four testable hypotheses follow from these considerations. (1) Repeated inflammation that does not reach the threshold of clinical episodes can progressively accumulate chromatin imprints in epidermal stem cells and lower the stimulus intensity required to trigger an episode; inflammatory memory in epidermal stem cells (10) and IFN-γ transcriptional memory (41) demonstrate that such imprints can be established and retained long-term, providing a mechanistic basis for this hypothesis. (2) Long-term interruption of the LL-37 positive feedback axis may partially reset local inflammatory programs; the interventional results of doxycycline, azelaic acid, and topical serine protease inhibitors indicate that the axis can be suppressed by available drugs (19–21). (3) Early intensive treatment may reduce relapse after withdrawal; early-intervention evidence in AD supports this direction (56–58). (4) Epigenetic and transcriptomic differences between lesional and non-lesional skin may serve as relapse-predictive biomarkers; stratification by resident T cells predicts early relapse after withdrawal in psoriasis (15), and serum cytokines rise at the time of clinical relapse (44), providing precedents for biomarker selection.

In terms of study design, we propose prospective cohorts with paired lesional and non-lesional samples collected during active disease and clinical remission, combining single-cell assay for transposase-accessible chromatin with high-throughput sequencing (ATAC-seq), histone modification profiling (for example, cleavage under targets and tagmentation or chromatin immunoprecipitation sequencing), DNA methylation arrays, and single-cell RNA sequencing to characterize candidate memory features, while simultaneously quantifying trigger burden and time to relapse after withdrawal. To test whether the TLR2/KLK5 axis encodes memory rather than merely reflects inflammation, future studies should combine TLR2 blockade or conditional genetic depletion in rosacea models and ex vivo human skin with longitudinal sampling across active and resolved disease, examining whether axis activity persists in clinical remission and predicts subsequent relapse. On this basis, biomarker-driven proof-of-concept RCTs could evaluate whether early intensive therapy or axis-blocking therapy extends the relapse-free period after treatment withdrawal.

## 9 Alternative explanations and contradictory evidence

The available evidence does not establish that inflammatory memory is the primary driver of chronic relapse in rosacea, and we deliberately weigh alternative explanations. Barrier dysfunction, microbial dysbiosis, and neurovascular dysregulation may be initiating factors, with the TLR2, mast-cell, and IL-1β signals discussed above representing downstream amplification or epiphenomena rather than memory-encoding mechanisms. The TLR2 and LL-37 axis evidence is largely correlational, based on expression analyses, transcriptomic profiling, and animal or ex vivo models; causal evidence in humans is limited to small interventional observations such as the effects of azelaic acid and topical serine protease inhibitors on axis activity and severity (20, 21). We therefore treat TLR2 overexpression as a candidate amplifier whose memory-encoding role remains unproven.

Contradictory or non-supportive findings must also be considered. The comparative histopathological study that found no difference in inflammatory mediator expression between mast cell-rich and mast cell-poor lesions (29) indicates that mast-cell abundance alone does not determine lesional inflammation. Single-cell data show resident memory T-cell populations in rosacea skin (38), but the infiltrates are sparse and the data are cross-sectional, so a bystander interpretation cannot be excluded. In addition, although non-lesional tissue can be re-primed ex vivo (32), this observation has not yet been reproduced longitudinally in patients, and its physiological relevance is unknown.

We therefore state the falsification criteria for the proposed model. The model would be contradicted if (1) no chromatin, epigenetic, or transcriptional memory mark persists in non-lesional or resolved skin during remission (2); depletion or inactivation of the proposed memory populations does not alter the risk of relapse (3); off-therapy remission can be fully explained by residual drug exposure or irreversible structural damage without any memory component; or (4) reduction of trigger burden fails to change relapse risk, despite evidence that triggers repeatedly induce episodes. Specifying these criteria is intended to make the framework falsifiable rather than to imply that it has already been confirmed.

## 10 Search strategy and selection criteria

This is a narrative review, and the literature was identified through structured searches of PubMed up to September 1, 2026, with targeted searches for each of the following theme groups (1): rosacea and mast cells, neurogenic inflammation, CGRP, TRPV1, MRGPRX2, or sensory nerves (2); rosacea and single-cell, transcriptomic, proteomic, multi-omics, or endotype studies (3); rosacea and T cells, tissue-resident memory, Th1/Th17, dendritic cells, or γδ T cells (4); rosacea and TLR2, TLR7, LL-37, cathelicidin, kallikrein, NLRP3, or inflammasome (5); rosacea and randomized trials, systematic reviews, guidelines, or network meta-analyses; and (6) rosacea and relapse, recurrence, trigger, flare, disease modification, remission, washout, or longitudinal outcomes. We included randomized clinical trials, cohort studies, case series, single-cell and histopathological studies, and mechanistic experiments in human tissue or relevant rosacea models, as well as the Cochrane systematic review, national guidelines, and network meta-analyses that could not be substituted by primary studies. Narrative reviews were not used as primary evidence sources; selected reviews were retained only for definitions and conceptual framework. Conference abstracts and studies not directly related to rosacea or to the reviewed mechanisms were excluded. Priority was given to primary literature published from 2023 to 2026, while irreplaceable mechanistic and clinical landmark studies published earlier were retained. In the revision-stage supplementary search, approximately 60 candidate titles and abstracts were screened and 37 records were bibliographically verified; together with the original search and iterative citation selection, this yielded the final list of 63 citations. Citation selection was iterative, driven by relevance to the inflammatory memory framework, chronic relapse, neuroimmune and mast-cell pathways, and disease modification.

## 11 Discussion and conclusion

Overall, **Figure 1** integrates the evidence reviewed above into a hypothetical multi-layer inflammatory memory model and outlines candidate intervention nodes for future disease-modification research. The inflammatory memory framework provides a unified mechanistic perspective on chronic relapse in rosacea, but the current evidence is largely indirect and direct molecular evidence is still needed. Disease modification is a worthwhile therapeutic goal, but quantitative relapse thresholds and disease-course endpoints must first be established. Moving from suppressing inflammation to rewriting tissue memory will require cross-scale basic and clinical research with long-term follow-up.

Future work should first answer whether memory traces exist in rosacea and then whether they can be rewritten. The former determines whether the framework holds, and the latter determines whether disease modification can become a realistic goal.
